# Response to: Need for longitudinal evidence on child and adolescent nutrition in the Amazon

**DOI:** 10.1017/S1368980026102766

**Published:** 2026-05-26

**Authors:** Rafael Martins da Costa, Edson dos Santos Farias, Marta Carolina Terto de Morais, Giovanna Eduarda da Silva, Geovane Biet de Sousa, Luis Gonzaga de Oliveira Gonçalves, Silvia Teixeira de Pinho

**Affiliations:** 1 Colegiado de Educação Física, Universidade Federal de Rondônia, Brazil; 2 Programa de Pós-Graduação em Psicologia, Universidade Federal de Rondônia, Brazil; 3 Programa de Pós-Graduação em Educação Física, Universidade Federal de Pelotas, Brazil; 4 Escola Superior de Educação Física e Fisioterapia, Universidade Federal de Pelotas, Brazil

**Keywords:** Adolescent health, Children health, Longitudinal studies, Paediatric obesity, Epidemiology

## Abstract

We welcome the Letter to the Editor and their thoughtful commentary on our recently published study examining temporal trends in overweight and obesity among schoolchildren in Porto Velho, Rondônia, Brazil, from 2006 to 2024. We fully concur with their central argument: the evidence base on child and adolescent nutrition in the Amazon region remains heavily reliant on cross-sectional and repeated cross-sectional designs, with longitudinal cohort studies representing a critical and largely unmet need. Our own findings underscore the urgency of building the longitudinal infrastructure necessary to identify causal determinants of these trends. The Amazon basin, home to approximately 40 million people across nine countries and over 300 indigenous ethnic groups, presents a uniquely complex socio-ecological context marked by rapid urbanisation, nutrition transition, environmental exposures and profound structural inequities that cross-sectional evidence alone cannot adequately characterise. We endorse the authors’ call for the prioritisation of long-term longitudinal studies and, in particular, their vision of multi-centre, transnational Amazonian cohorts supported by multi-country funding. We further propose that our established school-based surveillance infrastructure in Western Amazonia could serve as a meaningful foundation for future binational or multi-site collaborative research. We invite the authors to explore this possibility and look forward to contributing to a broader, coordinated research agenda aimed at improving child and adolescent health across the Amazon basin.

Dear Editor,

We are sincerely grateful to the authors of the Letter to the Editor for their thoughtful and substantive engagement with our recent publication. Their commentary not only reflects a careful reading of our work but also advances an argument we consider both timely and urgently necessary: the need for longitudinal evidence on child and adolescent nutrition across the Amazon basin.

We are especially encouraged to learn that the authors are currently conducting research on the nutritional status of children in Alto Amazonas, in the Peruvian region of Loreto. The fact that their project, comprehensive as it is, remains constrained to a cross-sectional design due to funding and logistical limitations is itself a powerful illustration of the very gap they identify and, we would argue, a shared challenge that unites researchers working in this region regardless of national borders.

We fully concur with the authors’ assessment that the existing evidence base from the South American Amazon relies heavily on cross-sectional or repeated cross-sectional designs. Our own study, which tracked temporal trends in overweight and obesity among 12 646 schoolchildren in Porto Velho, Rondônia, over 16 time points spanning 2006–2024, was conducted under precisely this methodological framework. Although the repeated cross-sectional approach allowed us to identify significant secular increases in obesity prevalence, particularly among girls (Annual Percent Change (APC) = 5·81 %; 95 % CI: 1·03, 10·81; *P* = 0·021) and children aged 6–10 years (APC = 5·20 %; 95 % CI: 1·17, 9·39; *P* = 0·017), we candidly acknowledge in our paper that the absence of longitudinal individual-level tracking constrains our ability to establish causal pathways or disentangle the complex biological, behavioural and socio-environmental determinants underlying these trends^(1)^.

The scarcity of longitudinal evidence that the authors document is striking, given the scale and complexity of the public health challenges facing Amazonian populations^(2)^. The Amazon is home to approximately 40 million people, including over 300 ethnic groups of indigenous peoples, many of whom contend simultaneously with poverty, food insecurity, limited access to health services and exposure to environmental threats such as mercury contamination from artisanal gold mining, a concern we specifically noted in the context of Porto Velho^(3,4)^. In this setting, cross-sectional snapshots, however methodologically rigorous, are insufficient to disentangle the trajectories of nutritional status over the life course or to identify the causal mechanisms that produce the accelerating obesity epidemic we document.

We wholeheartedly endorse the authors’ call to action for researchers across the Amazon to prioritise, wherever feasible, the design and establishment of long-term longitudinal studies. We further welcome and strongly support their vision of multi-centre Amazonian cohorts spanning multiple countries. The populations living across the Amazon basin, while exhibiting remarkable ethnic, cultural and linguistic diversity, share important structural, environmental and social dimensions of life that make comparative, transnational longitudinal research not only scientifically compelling but practically feasible. A multi-country cohort, ideally supported by multi-country funding mechanisms, could yield insights into the interplay between shared obesogenic pressures, such as the rapid nutrition transition towards ultra-processed foods and increased sedentary behaviour, and the context-specific factors that shape how these pressures manifest differently across national, geographic and cultural subgroups^(5)^.

We particularly appreciate the authors’ nuanced framing of the logistical and ethical challenges that complicate the implementation of longitudinal research in this context. Geographical inaccessibility, sparse and mobile populations, linguistic diversity and the imperative of meaningful engagement with Indigenous authorities and ethics committees are real and non-trivial barriers^(6)^. Our own experience in conducting systematic school-based anthropometric surveillance across the vast territory of Porto Velho, the largest capital city in Brazil by land area, has given us direct insight into the administrative, logistical and relational complexity of sustaining data collection over nearly two decades. These hard-won infrastructural assets represent a potential foundation upon which future collaborative, longitudinal work could be built.

In that spirit, we would be delighted to explore the possibility of a research partnership with the authors’ team. The combination of our ongoing surveillance infrastructure in the Western Brazilian Amazon and their current work in Alto Amazonas, Loreto, could serve as a natural starting point for the kind of binational, multi-site longitudinal collaboration the letter envisions. We believe that Porto Velho and Alto Amazonas, despite being separated by a national border, share enough socio-ecological features, such as rapid urbanisation, nutrition transition, environmental vulnerabilities and historical underinvestment in public health infrastructure, to make a comparative longitudinal design scientifically well-justified and particularly informative for regional policymakers on both sides of the border.

We echo the authors’ closing sentiment without reservation: longitudinal research is essential to disentangle the causal mechanisms driving child and adolescent nutritional outcomes in the Amazon and to inform effective, context-specific interventions. We commend the authors for raising this agenda in a high-visibility forum and for providing a comprehensive mapping of existing longitudinal evidence in the region. We hope this exchange will catalyse broader collaboration among the growing community of researchers committed to improving child health across the Amazon basin. We look forward to the possibility of continued dialogue.

## Data Availability

Access to the data can be made available upon request by the authors.
